# Role of EC-SOD Overexpression in Preserving Pulmonary Angiogenesis Inhibited by Oxidative Stress

**DOI:** 10.1371/journal.pone.0051945

**Published:** 2012-12-20

**Authors:** Shahana Perveen, Hardik Patel, Arslan Arif, Sharif Younis, Champa N. Codipilly, Mohamed Ahmed

**Affiliations:** Division of Neonatal-Perinatal Medicine, Cohen Children’s Medical Center of New York and Lilling Family Research laboratory, Feinstein Institute for Medical Research, North Shore-Long Island Jewish Health System, Manhasset, New York, United States of America; The Ohio State Unversity, United States of America

## Abstract

Angiogenesis is one of the most important processes for normal lung development. Oxidative stress can impair the pulmonary angiogenesis, leading to chronic lung disease or Bronchopulmonary dysplasia (BPD).

**Objective:**

To investigate the protective effects of EC-SOD overexpression on pulmonary angiogenesis on neonates following exposure to acute hyperoxia.

**Design/Methods:**

Transgenic (TG) and wild-type (WT) neonatal mice (10 mice per group) were exposed either to air (control group) or 95% O_2_ for 7 days starting at birth. After exposure, all animals were sacrificed. ROS concentration was measured in lung homogenates using OxiSelect ROS assay kit. Mean vascular density (MVD) was measured using anti CD34 staining. RNA was extracted and the angiogenesis markers, VEGF, VEGFR1 and VEGFR2 and PECAM-1 were analyzed by RT-q PCR. VGEF protein was measured using Western blots. Endothelial progenitor cells (EPCs) was assayed by flow cytometer.

**Results:**

There was a significant reduction of ROS in TG hyperoxic neonate group (156±14.2) compared to WT hyperoxic animals (255±35.1). Evaluation of MVD, using anti-CD34, showed marked significant increase of MVD in the TG group following hyperoxic exposure (85±12) in comparison to the WT hyperoxic group (62±8.4), (P<0.05). Among the hyperoxic groups, both RNA and protein of VEGF expression were significantly reduced in the WT animals compared to the TG group (P<0.05). The same trend was found in VEGFR 1 and 2 which were significantly reduced in WT group compared to the TG group (P<0.05). There was no significant difference between hyperoxia TG and control group (P>0.05). PECAM expression was significantly reduced in both hyperoxic compared to normoxic groups (P<0.05). EPC’s showed significant reduction in WT hyperoxic group compared to others (P>0.05).

**Conclusions:**

EC-SOD plays a key role in preserving angiogenesis by scavenging free radicals which has an inhibitory effect on angiogenesis process in neonatal mice lung following exposure to hyperoxia.

## Introduction

Angiogenesis, the growth of new capillary blood vessels from established vessels, is a complex phenomenon that leads to new blood vessel formation. The vascular growth involves endothelial cell migration, proliferation, differentiation, as well as tube formation [1]. Angiogenesis is a key process involved in normal development and is stimulated by variety of growth factors such as vascular endothelial growth factor (VEGF) [2]–[4]. Hypoxia triggers secretion of VEGF, which acts to increase the availability of oxygen from capillaries by increasing permeability and inducing formation of new blood vessels [5]. The spontaneous production of reactive oxygen species (ROS) by endothelial cells [6] is augmented by hypoxia/reoxygenation [7], [8] and by cytokines [9], [10]. ROS have been implicated in both lung [11] and vascular endothelial cell damage [12]. The fact that ROS are produced by endothelial cells, especially under conditions of re-oxygenation, and the very high sensitivity of endothelial cells to ROS poses a physiological need to scavenge these toxic oxygen radicals, which otherwise would lead to damage and apoptosis of the cells of the vasculature [12].

Strategies to reduce levels of ROS have emerged as a promising approach to treat conditions associated with enhanced oxidative stress. Scavenging of ROS is performed by a group of superoxide dismutases (SODs), enzymes which catalyze the dismutation of superoxide to hydrogen peroxide and oxygen. Endogenous EC-SOD is required for reparative neovascularization in response to ischemic injury by preventing overproduction of superoxide [13].

Extracellular superoxide dismutase (EC-SOD), is found throughout the vessel wall in many species and is the only isoform of SOD that is located outside of the cell, binding to cell surfaces and the extracellular matrix via its heparin-binding domain (HBD) [14], [15].

EC-SOD knocked out mice had increased sensitivity to lung injury, increased endothelial dysfunction and impaired neovascularization [4], [16], [17]. However, the functional importance of EC-SOD in vascular formation and function in the neonate stage is unknown.

It has been reported in premature neonates with bronchopulmonary dysplasia (BPD) that hyperoxia leads to disordered lung vascular development and marked decreases of VEGF levels compared to those premature neonates without BPD [18]. It was also speculated that hyperoxia suppression of VEGF signaling may be one of the mechanisms by which alveolarization can be arrested [19].

There are data to support the role of EC-SOD in improving vascular function among mature rats, and that it plays an important role in protection against endothelial dysfunction during aging [20]. Therefore, we hypothesized that overexpression of EC-SOD would reduce superoxide levels and improve angiogenesis in the neonatal lung exposed to hyperoxia.

## Materials and Methods

### Mice Model

All experiments involving animals were reviewed and approved by the Institutional Animal Care and Use Committee of the Feinstein Institute for Medical Research. C57BL6 mice Neonates wild-type (WT) and transgenic (TG), expressing extra copy of hEC-SOD under the β-Actin promoter [21] were housed in a pathogen-free environment, under standard light and dark cycles. An animal hyperoxia chamber system (BioSpherix, Lacona, NY, USA) was used for the *in vivo* studies. With this system, a constant 95% normobaric hyperoxia was achieved for 7 days (7d) in our study.

Studied animals were divided into four groups (ten per group) and housed for 7 d as follows: Group 1: WT neonate mice were exposed to hyperoxia at 95% oxygen. Group 2: TG neonate mice were exposed to hyperoxia at 95% oxygen. Group 3: WT neonate mice were housed at room air (RA). Group 4: TG neonate mice were housed at RA.

After exposure, pups were sacrificed; one lung/animal was fixed and inflated with formalin to be used for immunostaining. The other lung was divided into two halves, one was, suspended in RNA buffer solution and used for RT-qPCR assays and the other half was flash frozen in liquid nitrogen for ROS assay.

### Reactive Oxygen Species (ROS) Concentration Assay

Lungs were homogenized and ROS concentration was measured using OxiSelect ROS assay kit (Cell Biolabs, San Diego, CA). The assay was performed as prescribed in the manufacturer’s instructions.

### Evaluation of Mean Vascular Density (MVD)

For quantitative evaluation of angiogenesis, lung tissue sections were immunostained with human CD34 monoclonal antibody (BioGenex, San Ramon, CA). At low power field (x40), tissue sections were screened, and five areas with the most intense neovascularization were selected. Micro vessel counts of these areas were performed at HPF (x200) by a blinded reader. Counting of the micro vessels was performed with a computer image analyzer. An automated micro vessels count/field was computed in each spot, and the mean micro vessels count of the five most vascular areas was taken as the MVD, which was expressed as the absolute number of micro vessels/HPF. The MVD was measured based on Weidner’s method [22].

### Reverse Transcription-qPCR (RT-qPCR)

Total RNA was extracted using RNeasy mini spin columns (Qiagen, Valencia CA) from frozen lung tissue in RNAlater solution (Ambion, Austin TX). Purity and quantity of total RNA was assessed by Nano drop (Thermo Scientific, Waltham MA). RT-qPCR was performed using Thermo scientific Verso SYBR Green 1 Step QRT-PCR kit (Thermo Scientific, Waltham MA). Primers sequences were designed as per table 1. Thermal cycling was performed using Light cycler 480 (Roche Applied Science, Indianapolis IN), programmed for cDNA synthesis at 50°C 15 min followed by Thermo-Start activation at 95°C 15 min. Amplification (40 cycles) was performed as denaturation at 95°C for 15 sec, annealing at 57°C for 30 sec, extension at 72°C for 30 sec. and was followed by a melting curve to ensure specificity of the amplification reaction. Relative quantification analysis was performed by light cycler 480 software 1.5. Beta actin gene amplification was used as a reference gene for each sample and all the results were expressed as a ratio of Cp value of target gene to Cp value of Beta actin.

**Table 1:** Primer sequences for RT-qPCR for selected genes.

Gene		Primer Sequence
**VEGF**	**Forward**	GGAGATCCTTCGAGGAGCACTT
	**Reverse**	GGCGATTTAGCAGCAGATATAAGAA
**VEGFR1**	**Forward**	ATTTGTGATTTTGGCCTTGC
	**Reverse**	CAGGCTCATGAACTTGAAAGC
**VEGFR2**	**Forward**	GTGACCAACATGGAGTCGTG
	**Reverse**	CCAGAGATTCCATGCCACTT
**PECAM-1**	**Forward**	GAGCCCAATCACGTTTCAGTTT
	**Reverse**	TCCTTCCTGCTTCTTGCTAGCT

### Western Blot Analysis

Lung tissues were frozen and homogenized using mortar and pestle in liquid nitrogen. Tissue powder was incubated in RIPA buffer (150 mM NaCl, 0.1% Triton X-100, 0.5% Sodium deoxycholate, 0.1% SDS, 50 mM Tris-HCl, pH 8.8) supplemented with halt proteases and phosphatease inhibitor cocktail (Thermo Scientific, IL, USA) for 30 minutes on ice. Tissue lysates were centrifuged 13000 rpm at 4°C for 15 minutes and supernatant were collected. Protein concentrations were measured using microplate BCA protein assay kit (Thermo Scientific, IL, USA). Proteins were separated by standard 12% SDS-PAGE using bio-rad mini protean (Bio-Rad CA, USA). Proteins were transferred on nitro cellulose membrane overnight at 4°C. Membranes were blocked by 5% non fat milk, and incubated with anti-VEFG antibody (A-20) (Santa Cruz Biotechnology, CA, USA) for 1 hr. protein were detected using HRP-labeled anti-rabbit secondary antibody (Santa Cruz Biotechnology, CA, USA).

### Endothelial Progenitor Cells (EPC) Detection

Endothelial Progenitor cells were isolated as described elsewhere [23]. Briefly, after hyperoxia exposure animals were sacrificed and lung and heart were exposed.Pulmonary circulation was flushed with PBS thoroughly in order to remove circulating cell. Lung tissue were collected and minced in small pieces and placed in digestion buffer (DMEM-F-12, 20U/ml DNase I, 2 mg/ml Collagenase-1 for 1.5 hr at 37°C.The lung tissue was filtered through 40 µm cell strainer. RBC were lysed using lysis buffer (150mM NH_4_Cl, 10mM KHCO_3_ 2mM Na2-EDTA).Cells were re-suspended again in staining buffer (PBS,1%BSA and 0.09% NaN_3_) and stained with anti-CD45 Pacific Blue (30-F11), anti-Sca-1 PE (D7) and anti-CD133 APC (315-2C11) antibodies (Biolegend CA, USA). EPCs were detected using FACSVerse multicolor flow cytometer (Becton, Dickinson and Company NJ, USA).

## Results

### Evaluation of ROS

**Figure 1:**
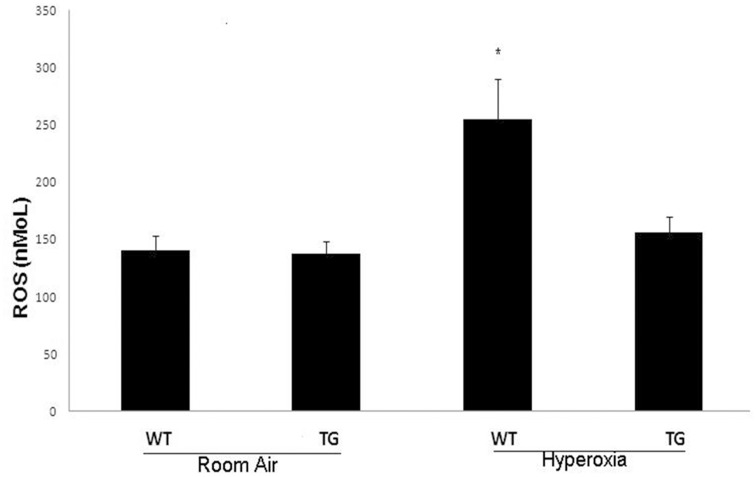
Evaluation of ROS concentration after exposure to hyperoxia (95% FiO_2_ for 7 days) in comparison to control air group. WT: Wild type. TG: Transgenic type. Data are mean of 10 animals/group ± SEM, * *P*<0.05.

There was a significant reduction of ROS in TG hyperoxia neonate group compared to the WT hyperoxia group (P<0.05). There was no significant difference between the TG hyperoxia and control normoxia groups (Fig. 1).

### Evaluation of MVD

**Figure 2:**
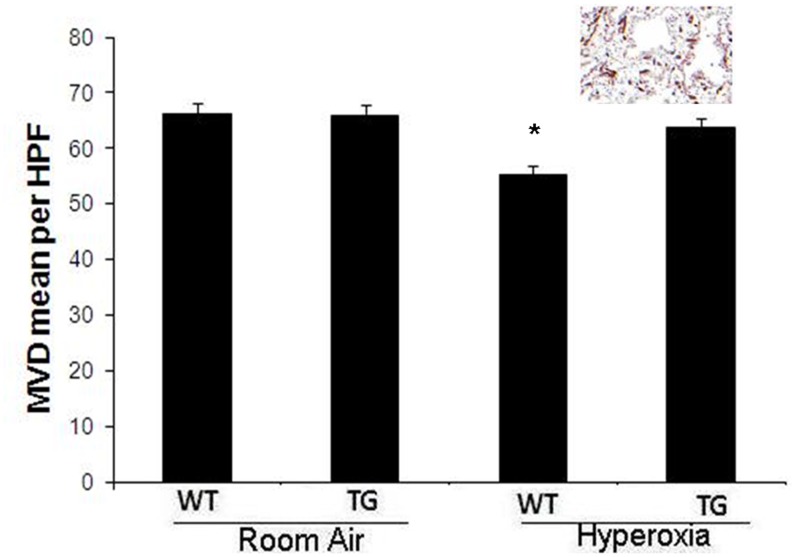
Evaluation of MVD by CD34 immunostaining in lung Formalin-fixed, Paraffin-embedded tissue in all groups after exposure to hyperoxia (95% FiO_2_ for 7 days) in comparison to control air group with insert of immunostaining. WT: Wild type. TG: Transgenic type. Data are mean of 10 animals/group ± SEM, * P<0.05.

Immunostaining with anti-CD34 showed a markedly significant reduction of MVD in the WT hyperoxic group with a mean of 62±8.4, in comparison to TG hyperoxic group with mean of 85±12 (P<0.05) (Fig. 2). There was no significant difference between the TG hyperoxic group and control room air groups.

### Angiogenesis Markers

**Figure 3:**
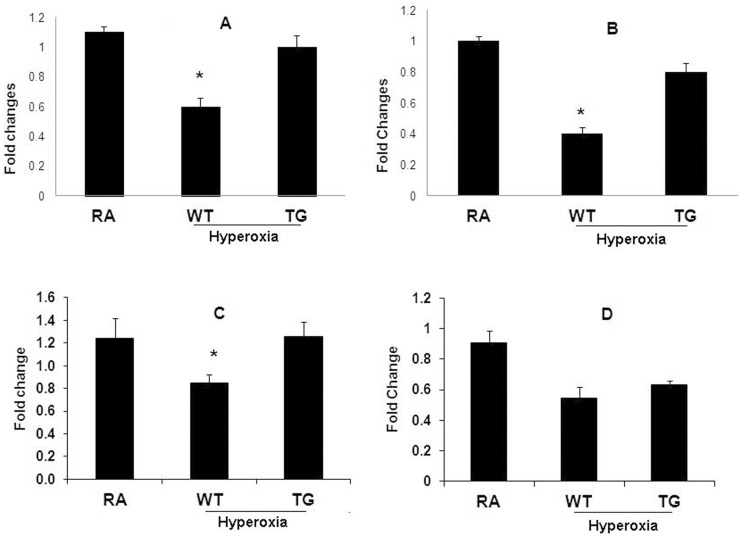
This panel demonstrates all RT-qPCR results in all groups after exposure to hyperoxia (95% FiO2 for 7 days) in comparison to control air group. A: RT-qPCR of VEGFR1 expression. B: RT-qPCR of VEGFR2 expression. C: RT-qPCR of VEGF. D: RT-qPCR of PECAM-1 expression. Data are mean of 10 animals/group ± SEM, * *P*<0.05.

RT-qPCR analysis showed both Vascular Endothelial Growth Factor Receptors (VEGFR) 1 and 2 expression were significantly reduced in the WT hyperoxic group compared to TG hyperoxic group (P<0.05) (Fig. 3A&B). Among hyperoxic groups, VEGF expression showed the same trend as VEGFR1&2, it was significantly reduced in WT group compared to TG group (P<0.05) (Fig. 3C). PECAM-1 expression was also significantly reduced in both hyperoxic groups (WT & TG) compared to normoxic groups (P<0.05), although there was no significant difference between WT hyperoxia and TG hyperoxia (P<0.05) (Fig. 3D).

**Figure 4:**
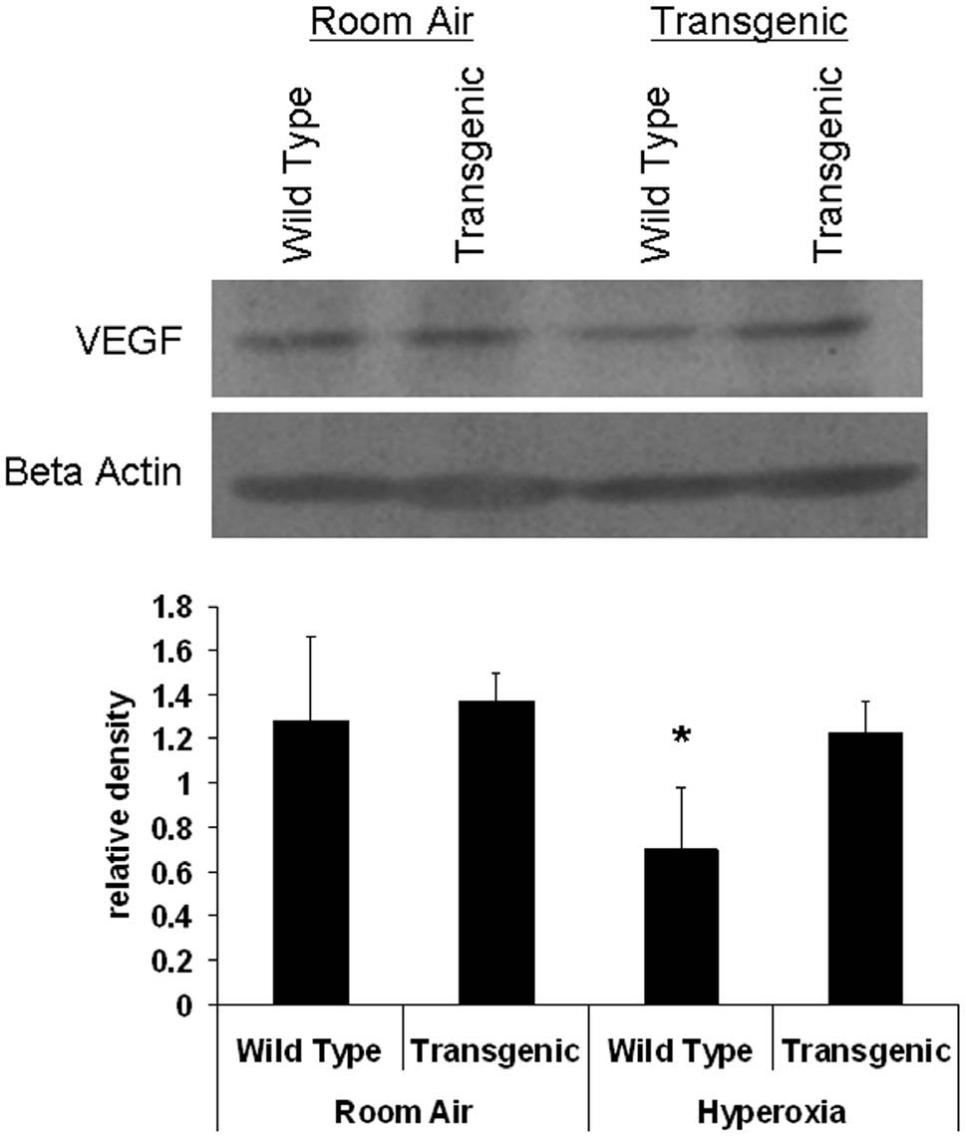
Western blot of VEGF in all groups after exposure to hyperoxia (95% FiO2 for 7 days) in comparison to control air group. VEGF expression was presented in relative density to Beta Actin expression. Data are mean of 5 animals/group ± SEM, * *P*<0.05.

VEGF protein level was reduced in WT hyperoxia animals compare to room air groups and level was restored in transgenic hyperoxia conditions (Fig. 4). These results complement RT-qPCR findings.

**Figure 5:**
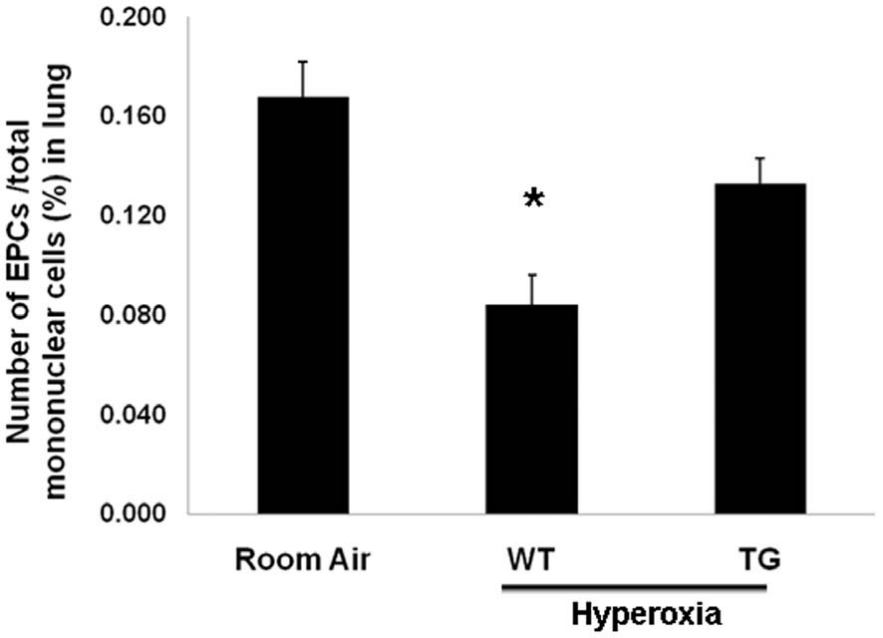
EPC’s Percentage by flow cytometer in all groups after exposure to hyperoxia (95% FiO_2_ for 7 days) in comparison to control air group. Data are mean of 5 animals/group ± SEM, * *P*<0.05.

EPCs were significantly reduced in hyperoxia wild type neonate mice compared to both RA and hyperoxia TG groups (P<0.05) (Fig. 5). There was no significant difference between hyperoxia TG in comparison to RA group.

## Discussion

Our study demonstrates the protective effect of EC-SOD over-expression on angiogenesis in neonatal lungs. We found that VEGF expression was significantly reduced in hyperoxic Wild type group compared to normoxic and hyperoxic TG groups (Fig. 3C & 4). A similar trend was noticed for VEGFR1 and VEGFR2 (Fig. 3A & B). Studying EPCs by flow cytometer showed a significant reduction in WT hyperoxia compared to TG hyperoxia and RA groups (Fig. 5). These findings potentiate the role of EC-SOD in protecting neonatal lung tissue against hyperoxia induced lung injury as we demonstrated in our previous study [24].

Studies involving de-activation of VEGF alleles [25] and knockouts of VEGFR–1 and VEGFR-2 receptor [26], [27], have shown the essential requirement of VEGF for development of the embryonic vasculature in mice. As shown in previous studies [19], there was a significant reduction in bothVEGFR1 and VEGFR2 mRNA expression after hyperoxia exposure compared to control animals [18]. Mice lacking EC-SOD expression showed impaired postnatal angiogenesis [28]. Also, it was found that administration of an anti–PECAM-1 leads to inhibition of endothelial cell migration, but not proliferation or survival, and impairs septation in neonatal rats, without reducing endothelial cell content [29]. Our data show after exposure to hyperoxia, there was loss of both VEGF and PECAM-1function which compromises postnatal lung development by inhibition of both endothelial cell function and alveolarization.

Several studies have demonstrated defective lung septation and emphysema in mice rendered deficient in VEGF, or where its receptor VEGF-R2 was blocked [30]–[33]. These findings suggest that VEGF signaling plays an important role in forming and maintaining normal alveolar structure during lung development [34]. VEGF treatment induces formation of new lung blood vessels, and this treatment improves lung development in an experimental model of chronic lung disease after premature birth [35]. In our study, we demonstrated a sparing effect of VEGF, VEGFR1and VEGFR2 expression in TG neonatal animals after exposure to hyperoxia compared to WT neonates. As angiogenesis in general and VEGF in particular, is necessary for alveolarization during normal lung development, it is reasonable to conclude that inhibition of VEGF during a critical period of lung growth contributes to the late sequelae of BPD. Furthermore, modulation of vascular growth factors may have therapeutic potential for lung diseases characterized by irreversible loss of alveolar structures [36].

Scavenging free radicals by EC- SOD is not the only mechanism by which EC-SOD can protect angiogenesis. VEGF-induced lung angiogenesis is in part mediated by nitric oxide (NO). Neonatal treatment with the VEGF inhibitor, down regulates endothelial NO synthase protein and NO production, and treatment with inhaled NO improves vascular and alveolar growth in this model of BPD [37]. Lungs of late fetal and neonatal endothelial NO synthase–deficient mice are more susceptible for failed vascular and alveolar growth after exposure to mild hypoxia and hyperoxia [38]. We did show before in a previous study, that over-expression of EC-SOD increases NO bioavailability to lung tissue in vitro and in vivo after exposure to hyperoxia stress [39]. This unique role of EC-SOD could explain another mechanism by which EC-SOD can protect the angiogenesis process in neonatal lung after exposure to hyperoxia.

Our data support the protective role of EC-SOD expression on angiogenesis as reported by Heistad [40]. Over expression of the human form of the enzyme protects mice from global cerebral ischemia [41], preserves post ischemic myocardial function [42], reduces lung injury during inflammation [43] and reduces aging induced cognitive impairment [44]. More recent studies indicate that the protective effect of EC-SOD on the oxidative fragmentation of the extracellular matrix component [45]–[47], is a key factor in controlling the inflammatory response in lung injury [48].

Animal models of bronchopulmonary dysplasia (BPD) and autopsy studies of humans who died from BPD have shown a reduction in the number of small arteries and an abnormal distribution of vessels within the distal lung [49], [50]. Endothelial progenitor cells (EPC’s) levels are also reduced in an experimental model of BPD in hyperoxic neonatal mice [23]. Hyperoxia is one of the leading and main factors which contribute to BPD development. Scavenging free radicals by overexpression of EC-SOD have multiple benefits by minimizing oxidant lung injury [24], [51], continuing lung development and keeping normal lung morphology [24] and prevent inhibitory effect on angiogenesis process as shown in our data (Fig. 5). All these findings enhance and magnify the role of EC-SOD in preventing BPD.

Thus, we conclude that overexpression of EC-SOD protects lung tissue against hyperoxia through a suppressive effect on ROS in neonatal lungs. This protective role preserves lung development, angiogenesis/neovascularization and alveolarization, which if compromised in early stages of development will lead to BPD. Therefore, future studies should examine the possibility of EC-SOD expression, or delivery of the protein or a mimetic as a means of protecting the developing lung exposed to hyperoxia.
